# The Maudsley model of anorexia nervosa treatment for adolescents and young adults (MANTRa): a study protocol for a multi-center cohort study

**DOI:** 10.1186/s40337-021-00387-8

**Published:** 2021-03-08

**Authors:** Tanja Wittek, Stefanie Truttmann, Michael Zeiler, Julia Philipp, Ellen Auer-Welsbach, Doris Koubek, Susanne Ohmann, Sonja Werneck-Rohrer, Petra Sackl-Pammer, Gabriele Schöfbeck, Dunja Mairhofer, Leonie Kahlenberg, Ulrike Schmidt, Andreas F. K. Karwautz, Gudrun Wagner

**Affiliations:** 1 Department of Child and Adolescent Psychiatry, Medical University of Vienna, 1090 Vienna, Austria; 2 Department of Neurology and Psychiatry of Childhood and Adolescence, Clinical Center Klagenfurt, 9020 Klagenfurt am Wörthersee, Austria; 3 Medical Practice for Child and Adolescent Psychiatry, 4020 Linz, Austria; 4 Institute of Psychiatry, Psychology & Neuroscience, King’s College London, London, WC2R 21S UK

**Keywords:** MANTRa, Maudsley model, Anorexia nervosa, Treatment, Psychotherapy, Adolescents, Young adults, Study protocol

## Abstract

**Background:**

The treatment of anorexia nervosa (AN) is often challenging due to medical complications as well as high relapse and mortality rates. Studies about effective treatment options for people with AN are particularly scarce in the adolescent population. This paper is a study protocol for a multi-center cohort study assessing the feasibility, acceptability and efficacy of a new, manualized treatment program, the “Maudsley Model of Anorexia Nervosa Treatment for Adolescents and Young Adults” (MANTRa) compared to psychotherapeutic treatment as usual (TAU).

**Methods/design:**

One hundred patients between 13 and 21 years who meet the inclusion criteria will receive 24 to 34 individual weekly MANTRa therapy sessions or weekly TAU sessions. Primary outcome variables will be BMI and eating disorder psychopathology 12 months after baseline. Further changes in central coherence, cognitive flexibility, emotion recognition, comorbid psychopathology (e.g. depression, obsessive-compulsive and anxiety disorders, non-suicidal self-injury), personality factors and therapeutic alliance will be assessed.

**Discussion:**

This multi-center study will examine the utility of the treatment program MANTRa for adolescents with AN and, therefore enhances the current knowledge about potential treatments for this patient group.

**Trial registration:**

ClinicalTrials.gov Identifier: NCT03535714. Registered: 24/05/2018 (retrospectively registered, still recruiting).

**Supplementary Information:**

The online version contains supplementary material available at 10.1186/s40337-021-00387-8.

## Introduction

Anorexia nervosa (AN) is a severe mental illness characterized by persistent restriction of energy intake, deliberate weight loss and, in further consequence, extreme low body weight [1]. Life-time prevalence among adolescents and young adults range between 0.5 and 1% with females being more affected than males [2, 3].

According to guidelines of the National Institute of Health and Care Excellence (NICE) [4] AN treatment needs to be multidisciplinary including medical care and weight monitoring as well as psychological interventions. Due to medical complications as well as high relapse and mortality rates treatment of AN is often challenging [5, 6]. Only half of all patients receiving AN treatment achieve full recovery after 4 to 10 years [7]. Recently published longitudinal studies showed that after 20 years of suffering from AN, recovery rates are still very low, ranging from 40 to 63% [8, 9].

Studies examining the efficacy of interventions for AN are particularly scarce in the adolescent population. A recent meta-analysis highlighted that there have been only eight published randomized-controlled trials (RCTs) that examined the efficacy of outpatient AN treatment for adolescents, with research dominated by studies comparing different versions of Family Therapy for AN (6 out of 8 studies) [10]. Family Therapy for AN, currently recommended as the only first-line psychological intervention for adolescents [4] has been found to significantly improve eating disorder (ED) psychopathology and weight gain; however, only 41.8% of the participants achieved full recovery at the end of therapy [11] and more than a quarter (26.6%) still suffered from AN after 2 to 4 years [12]. A study from Eisler and colleagues [13] showed significant differences of improvements in AN symptoms in favor of Multi-Family Therapy compared to Single-Family Therapy, possibly due to the fact that relationship patterns become more visible in the multi-family setting [14], but the percentage of patients having a good outcome after 1 year (measured with the Morgan-Russell scale) was limited in both groups ranging from 33 to 45% [13].

Enhanced Cognitive Behavioral Therapy (CBT-E) and Adolescent-Focused Therapy (AFT), the only recommended second-line psychological interventions for adolescents so far [4], have been found to significantly improve ED psychopathology as well as BMI [11, 15], but only 71.4% of patients receiving CBT-E fully completed the treatment while the remaining patients either required additional interventions or dropped-out [15]. Craig and colleagues [16] examined the use of CBT-E in an adolescent sample suffering from AN or atypical AN either as second-line treatment for patients after having received Family Therapy or in cases where Family Therapy was not applicable. They found significant improvements in ED psychopathology, but also a high attrition rate of 38.9% among those who started CBT-E. Altogether, the high rate of non-responders or treatment attrition in the established interventions as well as the lack of research emphasizes the need for the development of new therapeutic approaches in the adolescent population with AN.

MANTRA (Maudsley Model of Anorexia Nervosa Treatment for Adults), a manualized individual treatment program specific for patients with AN was developed by Schmidt and colleagues [17]. MANTRA’s efficacy has been examined in adults in several studies showing not only significant and stable improvements in ED psychopathology and BMI, but also high treatment acceptability and low drop-out rates [18, 19]. Consequently, MANTRA has been recommended as first-line psychological intervention for adults with AN [4]. This new intervention is centered around a patient’s workbook combining elements of cognitive behavioral therapy (e.g. a diagrammatic case formulation focusing on maintaining factors) and writing tasks, supporting emotion expression and regulation, based on the seminal ideas of James Pennebaker [20]. The therapeutic style of motivational interviewing (MI) is used to increase intrinsic motivation for change and overcome resistance [21].

The theoretical basis of MANTRA is the Cognitive-Interpersonal Maintenance Model of AN [22, 23] which considers predisposing personality traits like obsessive-compulsive and anxious-avoidant traits to be key vulnerability factors enhancing the risk of developing AN and contributing to the maintenance of the illness. Other factors known to maintain the illness and considered in MANTRA are: (1) impairments in the socio-emotional domain like the avoidance of emotions and reduced skills in social processing, (2) an inflexible, perfectionistic and detail-orientated thinking style, (3) pro-anorexia beliefs (positive beliefs about the value or function of the illness or particular symptoms, like ‘AN keeps me safe’ or ‘AN gives me control’) and (4) unfavorable communication styles of parents or close others. In contrast to CBT-E, weight and shape concerns are not considered as the central psychopathology of the disorder, but as symptoms of more profound and troubling problems [24].

Although MANTRA has not been applied to adolescents with AN so far, some of its characteristics might be especially beneficial for adolescents [25]: (1) MANTRA is centered around a patient’s workbook that every patient receives at the beginning of the treatment (for more details see ‘2.4.1 Intervention Group’). The therapist and the patient jointly select themes and exercises from the workbook based on the patient’s needs and symptomatology without a predetermined order. Patients are encouraged to read and perform additional exercises at home. The workbook offers the possibility to deal with therapeutic content outside the weekly therapy sessions, which could further improve treatment outcome [26]. For those not willing to complete homework outside the therapy sessions, exercises can exclusively be covered within the therapy sessions. (2) MANTRA is also conceptualized as a flexible and adaptive treatment program, in which patients are directly involved in the therapeutic process. Patients are encouraged to actively select topics which they want to work on. We assume that the possibility of taking responsibility and making active decisions during the therapeutic process could enhance motivation and self-efficacy which is very important for adolescents, as they strive for autonomy and independence during their normal pubertal development. (3) Additionally, one of the main targets of MANTRA is to enhance the patient’s intrinsic motivation for recovery. The empathic, respectful and accepting therapeutic style of MI aims to overcome resistance and gently guide patients towards recovery. Together, this helps to improve treatment engagement. (4) Another key element of MANTRA are writing tasks. People with AN often experience increased emotionality but at the same time have poorer awareness of emotions and less adaptive emotion regulation strategies than healthy controls [27]. We assume that especially young people with AN could benefit from expressing their thoughts and feelings in writing rather than verbally. Writing tasks may not only allow adolescents to take a broader perspective on their illness, but may also foster cognitive restructuring, reduce emotional avoidance and help generating new solutions [28]. (5) Another characteristic of the MANTRA workbook is the chapter focusing on identity. The ego-syntonic nature of AN and the high value of certain aspects of the illness (e.g. pro-anorexic beliefs) might be particularly relevant during adolescence and emerging adulthood, a developmental stage characterized by identity exploration and self-focus. In MANTRA, role models are used to construct alternative, non-illness-driven beliefs and values and enhance identity exploration and formation [29].

However, it is unclear whether the high acceptability, low dropout rates or improvements in ED psychopathology observed in adult patients receiving MANTRA [18, 19] can be transferred to adolescents with AN. This paper is a study protocol for a multi-center cohort study assessing the feasibility, acceptability and efficacy of an adapted version of the MANTRA treatment program for adolescents and young adults with AN (subsequently named “MANTRa”) compared to psychotherapeutic treatment as usual (TAU). We hypothesize that patients in the MANTRa intervention group will show a BMI increase and a reduction of ED symptoms at 12 months compared to baseline and that MANTRa will be superior to TAU with regard to BMI increase, ED psychopathology and treatment acceptability.

## Materials and methods

### Study design

The trial described in this study protocol paper is a multi-center cohort study, which will evaluate the efficacy of MANTRa (*n* = 50) compared with TAU (*n* = 50) in a sample of adolescent and young adult outpatients with AN. Assessments will be conducted prior to intervention start (baseline, T0), at 6-month (T1), at 12-month (T2) and at 18-month follow-up (T3). Researchers who will not be involved in conducting the medical and psychotherapeutic ED treatment will assess the outcome variables.

Ethical approval was obtained from the Ethics Committee of the Medical University of Vienna (EK 2005/2017). The data collection in this study follows the ethical standards laid down in the 1964 Declaration of Helsinki and its later amendments. The trial was registered at clinicaltrials.gov (Identifier: NCT03535714).

### Study setting, recruitment

This trial will be mainly conducted at three clinical centers in Austria: (1) The Eating Disorder Unit at the Department of Child and Adolescent Psychiatry, Medical University of Vienna, (2) the Department of Child and Adolescent Psychiatry, Medical University of Innsbruck and, (3) the Department of Neurology and Psychiatry for Children and Adolescents of the Clinical Center Klagenfurt. Further patients will be recruited from the “Sowhat Competence Center for Eating Disorders” in Vienna and cooperating child and adolescent psychiatrists in medical practices.

Recruitment for the trial started in April 2018 and is currently on-going. We expect to reach the final sample size in January 2021. Data collection will be completed in July 2022.

### Eligibility criteria

All patients who will receive ED treatment at one of the clinical centers or medical practices will be asked to participate in the study if they meet the inclusion criteria. Patients will be eligible if they:

- are aged between 13 and 21
- are diagnosed with full-syndrome AN, atypical AN or weight restored AN (following inpatient treatment) according to DSM-5
- are willing to engage in psychotherapy

Patients will be excluded if they:

- suffer from life-threatening AN requiring immediate inpatient treatment as defined by the German guidelines for diagnosis and treatment of eating disorders [30]
- have insufficient cognitive abilities or German language skills to follow the treatment content
- suffer from a severe mental or physical illness that needs priority treatment (for example psychosis, acute suicidality, borderline personality disorder, substance abuse)
- are pregnant

The eligibility criteria will be checked in appointments (called “Clearing phase”) by the case manager (psychologist, psychiatrist). After checking the eligibility criteria, potential trial participants and their legal representatives will be informed about the study procedures by child and adolescent psychiatrists and/or psychologists not involved in the MANTRa or TAU psychotherapy either in face-to-face meetings or via telephone including the option to ask questions. Written informed consent from one of the legal representatives (if aged below 18) and the patient’s assent will be obtained prior to the inclusion in the study.

### Interventions

MANTRa will be compared to a TAU group covering different non-manualized psychotherapeutic approaches for the treatment of AN. Thus, the TAU group will represent the landscape of currently available psychotherapeutic treatments for adolescent AN in Austria. Therefore, the study does not aim to contrast the MANTRa intervention to another specific therapeutic intervention but to explore its acceptability and efficacy compared with psychotherapeutic interventions that can be regarded as standard care in Austria (e.g. cognitive-behavioral therapy, systemic family therapy, psychodynamic or psychoanalytic therapy or humanistic approaches).

#### Intervention group (MANTRa)

The MANTRa intervention (‘Maudsley Model of Anorexia nervosa treatment for adolescents and young adults’) is an adapted version of the original MANTRA treatment program for adult patients with AN and is designed as an outpatient manualized treatment program consisting of 20 weekly therapy sessions of approx. 50 min. followed by 4 monthly booster sessions. Patients with a more severe illness (one or more comorbidities, BMI below the 3rd sex and age-specific BMI percentile and illness duration longer than 1 year) will receive an extended version consisting of 30 weekly sessions, followed by 4 booster sessions. Whether a patient will receive the longer or shorter version will be decided according to the criteria mentioned above prior to the intervention start.

The original workbook [17] was translated into German and had been adapted to the developmental level of adolescents in terms of language, design and content by our research team. A tabular description of the differences between MANTRA for adults compared to MANTRa for adolescents according to the TIDieR framework [31] is provided as supplementary material. For example, explanations for technical terms were inserted and a form of address customary for young people was used. In terms of content, we created three case vignettes representing typical adolescents suffering from AN, which are introduced in the first chapter and occur in every chapter explaining certain exercises. The case vignettes enable the patient to identify with adolescents who experienced similar events and who could serve as role models on the way to recovery. In chapter four, we changed nutritional recommendations according to suggestions of our dieticians who are experienced in the field of adolescent’s EDs (e.g. portion sizes are illustrated using the hand proportions and fists, not focusing on calories) and added information and exercises for managing binge eating and purging behavior. Dietological exercises of chapter four, such as the creation of a meal plan and nutritional recommendations are carried out by dieticians (see section ‘2.4.5. Relevant concomitant care permitted during trial’). BMI tables were replaced by BMI-percentile graphs. In chapter seven, which addresses emotions, we added the distress tolerance exercises from the Dialectic-Behavioral Therapy for Adolescents [32], because adolescents suffering from AN often engage in non-suicidal self-injury behaviors or experience emotion regulation problems [33]. Thus, adolescents need effective tools to help dealing with and expressing overwhelming emotions, as well as understanding the emotion regulation function of AN [34]. We also added a new chapter about social media and mobile applications to the workbook which was based on materials provided by South London and Maudsley NHS Foundation Trust [35], because social media platforms provide the opportunity and risk to communicate and exchange illness related as well as health-related thoughts known to be frequently used by adolescents [36]. Discussions about helpful and unhelpful aspects of social media platforms can be important for adolescents. Furthermore, we also adapted the design and layout of the workbook to make it more appealing for adolescents by creating each chapter in a different color and adding icons for better visual recognition of recurrent content (e.g. a letter icon for writing tasks or a question mark for reflection exercises). An overview of the chapters of the adolescent version of the workbook can be found in Table 1.

**Table 1:** Chapters of the MANTRa patient’s workbook

Chapter	Title	Content / Aim
1	Introduction to MANTRa	Introduction to MANTRa, description of setting and treatment efficacy, case vignettes
2	The journey begins	Enhancing motivation for change, pro and cons of the illness
3	Nobody is an island – support from others	Social environment, families, support through others
4	Physical health and nutrition	Physical health, healthy nutrition, meal plans, dietary
5	My Anorexia	Case formulation, causing and maintaining factors of the illness, vicious flower
6	Treatment goals	Developing treatment goals, SMART goals
7	Emotions and social relationships	Emotion recognition and regulation skills, social relationships and roles, the inner critic voice, self-compassion
8	Thinking styles	Perfectionism, detail-orientation, rigidity and inflexibility
9	Social media	Social media as maintaining factor of the illness
10	Identity	Role models, identity with and without anorexia
11	The flower of life	Relapse prevention, virtuous flower

Similar to the original MANTRA program, close others will be invited to participate in 2 to 3 MANTRa sessions if necessary. The aim of this joint sessions is to discuss conflicts within the family and to develop strategies how close others can support the patient, thus promoting a common understanding of the disease and ways to recovery. Additionally, parents will be included regularly in the medical treatment provided by the case manager and will be invited to participate in the parental skills training “SUCCEAT” (see section ‘2.4.5. Relevant concomitant care permitted during trial’) [37–39].

We recruited clinical psychologists or licensed psychotherapists specialized in cognitive-behavioral therapy from the Department for Child and Adolescent Psychiatry at the Medical University in Vienna or from the Austrian Society for Behavioral Therapy as study therapists. In total, 13 clinical psychologists or psychotherapists agreed to participate. They will conduct the MANTRa intervention with an estimated 1 to 4 patients each in their private practices or in their clinic offices. Beforehand, all therapists attended a 2-days MANTRa training workshop offered by U. Schmidt as well as a 1.5-day MI workshop offered by a licensed MI-trainer. Regular team meetings to discuss cases will be organized by the research team and therapists will be encouraged to participate in MANTRa group supervision provided by U. Schmidt. The clinician engagement in group supervision will be recorded by the research team. MANTRa therapist will only treat patients in the intervention group.

#### Treatment as usual group (TAU)

Patients in the TAU group will receive outpatient psychotherapy from psychotherapists experienced in EDs, who are licensed as psychotherapists according to the Federal Ministry of Social Affairs, Health, Care and Consumer Protection. Psychotherapeutic approaches of the TAU group cover different, approved methods, like cognitive-behavioral therapy, systemic family therapy, psychodynamic or psychoanalytic therapy or humanistic approaches (e.g. gestalt therapy, logotherapy and existential analysis, etc.). Case managers will suggest a suitable psychotherapist from a pool of AN experienced psychotherapists and will organize an initial treatment appointment in cooperation with the parents. This is the standard procedure for the allocation of patients to psychotherapy in the Austrian health care system, where psychotherapy is mostly provided by external, individual practitioners.

#### Assignment of intervention

Patients undergoing treatment at the Department of Child and Adolescent Psychiatry in Vienna who will not attend any other psychotherapy or psychological treatment or have discontinued their psychotherapy prior to the baseline assessment, will be assigned to the MANTRa intervention group. Patients who will be treated in the other centers or who have already recently started psychotherapy will be assigned to the TAU group. The allocation of patients to the therapists will be based on availability and local proximity in both groups.

#### Adherence to the intervention and evaluation

MANTRa is designed for flexible use (e.g. no predetermined order in which MANTRa workbook has to be worked through, modules may be omitted if not relevant for a specific patient). In order to assess general adherence to the MANTRa intervention, therapists will be asked to keep a written record of the main topics discussed / modules of the workbook addressed in each session. Additionally, the 8th and 16th therapy sessions will be audiotaped. The TAU group should reflect a representative treatment-as-usual setting; therefore, no specific regulations or strategies to assess adherence will be implemented.

In both groups, the therapist discusses the general conditions and the groundrules of psychotherapy in the first session, including procedures in case of short-term cancellation of appointments, illness, confidentiality, data protection, etc. In case of unexcused absence, the therapist contacts the patient to arrange a new appointment. In case of repeated cancellations, this will be discussed in the therapy with the patient in order to increase adherence.

In order to promote participant retention in research assessment, each participant will be invited personally by the research team. In both groups, patients will be contacted twice via telephone and afterwards once or twice via text message/email. If participants do not respond, their parents will be contacted to establish contact with the participant. Reminders will be sent if questionnaires have not been completed in time. Furthermore, case managers are committed to this project and will further motivate the patients to participate in follow-up assessments.

#### Relevant additional care permitted during trial

In both groups, participants will be in regular medical care to monitor the physical health and weight gain provided by a case manager (child and adolescent psychiatrists or child and adolescent psychiatrists in training under supervision, psychologist), and will receive nutritional consultation by a dietician. Parents will be regularly included in the medical treatment provided by the case manager and will additionally be invited to participate in the parental skills training “SUCCEAT” which aims to reduce caregiver burden and supports parents in caring for a person with an ED [37–39].

During the course of this trial, the study participants will not be allowed to attend two different forms of individual psychotherapeutic treatment simultaneously. Participants will be encouraged to continue psychotherapy or start a new psychotherapy if they are still suffering from AN or another mental disorder after MANTRa or TAU had ended. Case managers will be responsible for ensuring appropriate follow-up treatment.

#### Criteria for discontinuing or modifying allocated interventions

AN symptomatology and medical risk will be monitored continuously by the case manager. MANTRa and TAU will be both suspended in case inpatient treatment becomes necessary due to worsening of AN symptomatology, i.e. further weight loss in an outpatient setting, as well as deterioration of medical parameters and/or somatic/psychiatric comorbidity. These criteria are in accordance with recommendations for starting inpatient treatment of the German guidelines for diagnosis and treatment of eating disorders [30]. After discharge, the intervention will be continued. Participants will be also allowed to ask for a change of the therapist, if the therapeutic relationship is experienced as not suitable for them.

### Outcomes

**Fig. 1:**
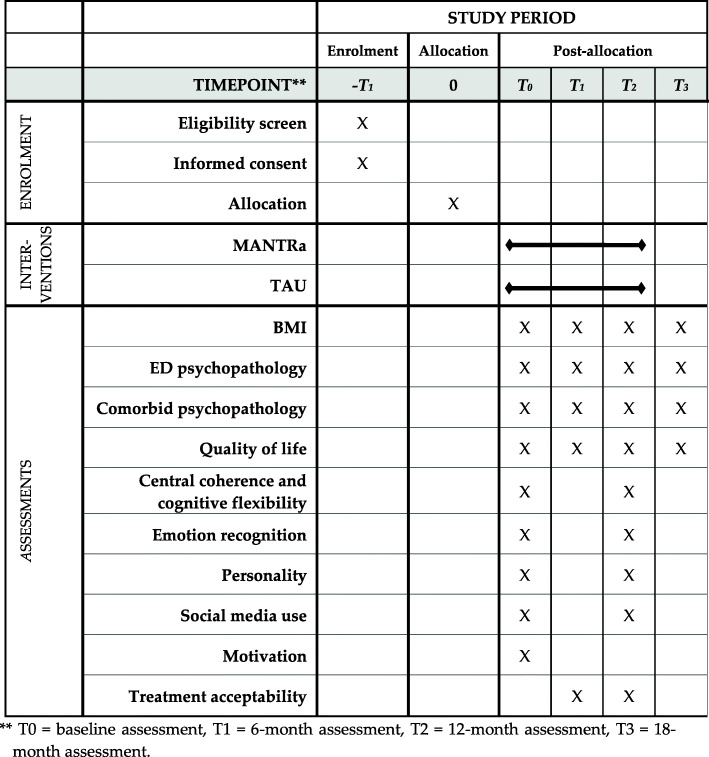
Schedule of enrollment, interventions and assessments. ** T0 = baseline assessment, T1 = 6-month assessment, T2 = 12-month assessment, T3 = 18-month assessment

All outcome variables will be assessed prior to intervention start at baseline (T0) and after 12 months (T2). The primary outcome (ED psychopathology), comorbidities and quality of life will be additionally collected 6 months after baseline (T1) and at 18-month follow-up (T3) (see Fig. 1 for details). Self-report questionnaires will be administered online via ‘LimeSurvey’, while interviews will be conducted face-to-face or via telephone by trained professionals.

#### Primary outcomes: eating disorder psychopathology

- Body Mass Index (kg/m^2^) at 12 months.
- Eating Disorder Examination (EDE) [40] global score at 12 months. The semi-structured EDE interview is gold standard in ED research. It comprises four subscales (dietary restraint, eating concern, weight concern, shape concern) and a global score assessing general ED psychopathology. The EDE has excellent reliability (total score: Cronbach’s α = .93); good convergent validity (.56 < r < .81) was observed with other measures of ED pathology [40]. Similar results were found in an adolescent population (Cronbach’s α = .93) [41]. Two psychologists trained and supervised in conducting the EDE interview by a senior clinical psychologist (G.W.) will perform the interviews. They will not be blind to the intervention but are part of the independent research team and not involved in the clinical treatment and psychotherapy.
- Eating Disorder Inventory (EDI-2) [42] global score at 12 months. The EDI-2 is a self-rating questionnaire assessing various ED dimensions through 11 subscales (Drive for thinness, Bulimia, Body Dissatisfaction, Ineffectiveness, Perfectionism, Interpersonal Distrust, Interoceptive Awareness, Maturity Fears, Asceticism, Impulse Regulation and Social Insecurity) and a total score. The measure has excellent internal consistency (total score: Cronbach’s α = .90).

#### Secondary outcomes

- Comorbid psychopathology: The State-Trait Anxiety Inventory (STAI) [43] is a self-rating questionnaire assessing anxiety. The STAI has excellent internal consistency (Cronbach’s α ≥ .89). Depression will be measured with the Beck Depression Inventory (BDI-2) [44], a self-rating questionnaire with excellent internal consistency (Cronbach’s α = .92) observed in an adolescent population [45]. The Obsessive-Compulsive Inventory Revised (OCI-R) [46] is a self-rating questionnaire for the assessment of obsessive-compulsive traits and symptoms. The German version demonstrated excellent validity and internal consistency (total score: Cronbach’s α = .86) for adults; no data are currently available for adolescents [46]. The STAI, BDI-2 and OCI-R will be completed by the participants. The presence of a comorbid psychiatric disorder (anxiety or obsessive-compulsive disorder, depression, non-suicidal self-injury, post-traumatic stress disorder) according to DSM-5 diagnostic criteria is diagnosed by a psychologist via a semi-structured clinical interview (Diagnostic Interview for Mental Disorders in Children and Adolescents; Kinder-DIPS) [47].
- Quality of life will be measured with the German version of the Inventory for Assessment of Quality of Life in Children and Adolescents (ILK) [48], a self-rating questionnaire with acceptable internal consistency (Cronbach’s α = .55–.76) measured in an adolescent population.
- Central coherence and cognitive flexibility: Computergestütztes Kartensortierverfahren (CKV) [49] is the German version of the Wisconsin Card Sorting Task which measures cognitive flexibility and set-shifting ability. So far, there is no information about the reliability of this measure, but the split-half reliability of the English version is excellent (.90 ≤ r ≤ .95) [50]. The Rey Complex Figure Test and Recognition Trial (RCFT) [51] is a neuropsychological drawing task measuring gestalt processing. The drawing will be rated by a psychologist according to the scoring system from Booth [52] in order to calculate a central coherence score. The inter-rater reliability ranges from .71 to .97.
- Emotion recognition: The Frankfurter Test und Training of Facial Affect Recognition (FEFA-2) [53] is a computerized neuropsychological task testing facial affect recognition showing good psychometric properties (Cronbach’s α ≥ .91). The Toronto-Alexithymia Scale-26 (TAS-26) [54] is a self-rating questionnaire measuring alexithymia, a personality trait characterized by the inability to identify and describe emotions experienced by oneself or others, which is often impaired in people with AN [55, 56]. Internal consistency examined in an adolescent sample is moderate to good (Cronbach’s α = .67–.84) [54].
- Personality: The subscales harm avoidance, persistence and self-directedness of the Junior Temperament and Character Inventory (JTCI) [57] will be used to measure perfectionism and rigid personality traits. The three subscales of this self-rating questionnaire for adolescents showed good internal consistency (Cronbach’s α = .82–.86).
- Social media use: We constructed a self-rating questionnaire based on questions of the Pro-Anorexia Website Survey (PAWS) [58] to assess the frequency of social media use and the influence on ED behavior prior and after the intervention.
- Motivation: Motivation for change will be measured with the Anorexia Nervosa Stages of Change Questionnaire (ANSOQC) [59], a self-rating questionnaire with good reliability (Cronbach’s α ≥ .76) and validity for adolescents [60]. The Psychotherapy Motivation Questionnaire (FPTM-40) [61] measures the motivation to attend psychotherapy from a patient’s perspective. The internal consist of this measure is acceptable (Cronbach’s α = .70).
- Treatment acceptability: Treatment acceptability will be assessed with the Questionnaire to Assess Treatment (FBB) [62], which has good internal consistency (therapist version: Cronbach’s α = .88; patient version: Cronbach’s α = .83). The therapeutic alliance as possible mediating factor will be with the self-rating questionnaire Therapeutic Alliance Scale for Children (FTB-KJ) [63]. The measure has good internal consistency (therapist version: Cronbach’s α = .83; patient version: Cronbach’s α = .82). Both, FBB and FTB-KJ will be filled in by the participants and the therapists.
- Treatment process and feasibility of the intervention: The Session Evaluation Questionnaire (SEQ-d [64] will be used in the MANTRa group for systematic examination of the treatment process. The SEQ-d will be completed after each session by the therapist and the patient (not listed in Fig. 1). The internal consistency of the English version is excellent (Cronbach’s α = .80–.92). There is no data about reliability or validity for the German version so far [65]. Additionally, we will use qualitative methods to gain insights into the patients’ and therapists’ personal experiences with the MANTRa therapy and to further improve the workbook: For example, we will analyze parts of the therapy content (formulation and goodbye letters, virtuous and viscous flower) by means of qualitative analyses. In addition, we will ask patients about their experiences with the MANTRa treatment program at T2 using 5 open-ended questions and we will interview the MANTRa therapists with semi-structured interviews. Qualitative data will be analyzed in NVivo software [66].

### Sample size

The sample size calculation was conducted with G*Power Version 3.1.9.2 and was based on the primary outcome analysis (difference in BMI, EDE total score and EDI total score between the baseline and 12-month follow-up assessment in the MANTRa intervention group). Based on previous studies, at least medium effect sizes (dz = 0.5) for the change in BMI and ED pathology can be expected [18, 67]. Using *t*-tests for paired samples, a sample size of 34 for the MANTRa group is needed to detect an effect size (dz) of 0.5 (alpha 5%) with a power of 80%. To account for a dropout rate of 25%, a total of 50 patients will be recruited for the MANTRa intervention group.

Furthermore, we plan to obtain the same number of patients (*N* = 50) for the control group. Sensitivity calculations revealed that differences in the primary outcome variables between the MANTRa and control group can be detected for small to medium effects (f = 0.14) (group x time interaction using general linear mixed models). Using multi-center recruitment, it is expected to reach the target sample size.

### Data management

Self-rating questionnaires will be completed by the participants online via ‘Limesurvey’. Data from this survey tool will be directly imported into IBM Statistics SPSS software afterwards. Interview data will be entered manually by the research team. A data quality check will be carried out to exclude implausible values before the statistical analysis. Personal information allowing patients’ identification will be stored separately from study data in order to protect confidentiality. Only authorized persons of the MANTRa research team will have access to the trial data.

### Statistical analysis

The primary statistical analysis will be based on the intention-to-treat principle, which means that participants will be analyzed in the group to which they were allocated irrespective of their compliance with the assigned treatment. Missing data will be imputed; the specific imputation model will be developed once the final data is available. *T*-tests for paired samples will be carried out for analyzing pre-post differences (T0-T2) in the three primary outcome variables (BMI, EDE, EDI-2). To account for multiple testing (three primary outcome variables) and the inflation of the Type I error rate, we will use Bonferroni correction to adjust the significance level (α = .017). Additionally, a completer analysis will be conducted as a secondary analysis. General linear mixed models will be used to measure effects between groups and over time (T0, T1, T2, T3) with using the treatment type (MANTRa vs. TAU) as between factor and assessment time (T0, T1, T2, T3) as within-factor. The treatment type x time interaction will indicate whether there is a difference in the course between the MANTRa and control group. Additional *t*-tests will be used to explore group differences between intervention (MANTR-a) and control group (TAU).

### Dissemination

Results will be presented at scientific conferences and published in peer-reviewed journals. A publication plan has been jointly developed by the MANTRa research team. Flyers presenting the results in simplified form will be prepared and sent to the participants and their parents if desired.

## Discussion

The aim of this study is to assess the feasibility, acceptability and efficacy of the manualized treatment program MANTRa compared to TAU in an adolescent sample suffering from AN.

A combination of medical care and psychological interventions are recommended for the treatment of AN [4], but data on effective psychological treatment methods are limited, particularly for adolescents [10]. High treatment attrition or non-response rates highlight that a sub-group of patients do not benefit sufficiently from existing therapies and further is at high risk of chronicity. Thus, it is even more important to develop and examine new treatment programs for adolescents.

The MANTRA treatment program is considered to be a first-line treatment method for adults suffering from AN [4], that has been found to effectively improve ED psychopathology [18, 19]. However, to date this treatment has not yet been applied to an adolescent population with AN, even though some characteristics could be especially beneficial for adolescents, like the use of MI to enhance intrinsic motivation for recovery, writing tasks to overcome avoidance of emotions, the flexible and participatory structure and the focus on identity development.

### Strenghts

This trial has several strengths, for example, different outcome variables are used to evaluate changes, not only including BMI and weight gain, but also ED symptomatology and neuropsychological variables. Additionally, potential moderators like comorbid psychiatric disorders, personality traits and therapeutic alliance are assessed to evaluate predictors for good/poor outcome.

In this study, we also include the broad spectrum of AN patients (full-syndrome AN, atypical AN, weight-restored AN) in our sample to increase the generalizability of our results for different stages of the illness.

Another big strength of our study is the long time period of 1.5 years between the baseline and follow-up assessments. As it often takes a long time for ED symptoms to change, it is very important to have long follow-up periods for examining the efficacy of an intervention.

### Limitations

A limitation of this paper is the absence of randomization. Instead, patients will be assigned to the group based on predefined criteria, like the presence of a recently started psychotherapy or centre of treatment (for details see 2.4.3 Assignment of intervention). This could lead to an assignment bias, where groups may differ in terms of therapeutic alliance at baseline. However, it would be unethical to disrupt an already existing therapeutic process unless the patient requests it. In order to minimize possible group differences at baseline, we will statistically compare both groups regarding clinical and sociodemographic characteristics (e.g. age, ED severity, educational level) and include them as covariates in the statistical analysis if necessary. Furthermore, group comparability may be limited due to the study settings (e.g., audio recordings and evaluation after the therapy session only in the MANTRa group). Thus, differences between MANTRa and TAU can only be interpreted as observational, not as causal. Nevertheless, the comparison with TAU may serve as a basis for a larger randomized controlled trial.

A sample size of *N* = 50 per group might also be too low to statistically detect small between-group effects; however, the planned sample size represents a reasonable number of patients who can be recruited and involved in the study within the planned time frame. Nevertheless, the planned sample size should be sufficient to explore the feasibility and acceptability of MANTRa compared to TAU as well as to explore the size of effects regarding the treatment success that can be expected. If the results of this study are promising, this information can be used to design a larger randomized-controlled trial.

## Conclusions

In summary, MANTRa could be an effective and helpful intervention not only for adults, but also for adolescents suffering from AN. This paper presents the protocol for a study examining the feasibility, acceptability and efficacy of this new treatment program and therefore enhancing the current knowledge about potential treatments for adolescent AN.

## Supplementary Information

**Additional file 1.** A tabular description of the differences between MANTRA for adults and MANTRa for adolescents according to the TIDieR (Template for Intervention Description and Replication) Checklist by Hoffmann and colleagues (2014)

## Data Availability

Not applicable.

## Abbreviations

AN: Anorexia nervosa
MANTRa: Maudsley Model of Anorexia Nervosa Treatment for Adolescents and Young Adults
TAU: Treatment as usual
NICE: National Institute of Health and Care Excellence
ED: Eating disorders
CBT-E: Enhanced Cognitive Behavioral Therapy
AFT: Adolescent-Focused Therapy
MANTRA: Maudsley Model of Anorexia Nervosa Treatment for Adults
MI: Motivational Interviewing
EDE: Eating Disorder Examination
EDI-2: Eating Disorder Inventory-2
STAI: State-Trait-Anxiety Inventory
BDI-2: Beck Depression Inventory-2
OCI-R: Obsessive-Compulsive Inventory Revised
Kinder-DIPS: Diagnostic Interview for Mental Disorders in Children and Adolescents
ILK: Inventory for the Assessment of Quality of Life in Children and Adolescents
CKV: German Version of the Wisconsin Card Sorting Test
RCFT: Rey Complex Figure Test and Recognition Trial
FEFA-2: Frankfurter Test and Training of Facial Affect Recognition
TAS-26: Toronto-Alexithymia Scale-26
JTCI: Junior Temperament and Character Inventory
PAWS: Pro-Anorexia Website Survey
ANSOQC: Anorexia Nervosa Stages of Change Questionnaire
FPTM-40: Psychotherapy Motivation Questionnaire
FBB: Questionnaire to Assess Treatment
FTB-KJ: Therapeutic Alliance Scale for Children
SEQ-d: German Version of the Session evaluation questionnaire
