# Supplementary material for: The Maudsley model of anorexia nervosa treatment for adolescents and young adults (MANTRa): a study protocol for a multi-center cohort study
Source: J Eat Disord. 2021 Mar 8;9:33. doi: 10.1186/s40337-021-00387-8 (PMC7941930; doi:10.1186/s40337-021-00387-8)
Supplement: Supplementary file 1 — Additional file 1. A tabular description of the differences between MANTRA for adults and MANTRa for adolescents according to the TIDieR (Template for Intervention Description and Replication) Checklist by Hoffmann and colleagues (2014) [file 40337_2021_387_MOESM1_ESM.docx]

**SUPPLEMENT:** A tabular description of the differences between MANTRA for adults and MANTRa for adolescents according to the TIDieR (Template for Intervention Description and Replication) Checklist by Hoffmann and colleagues (2014)

| Item Nr. | Item | MANTRA for Adults | MANTRa for Adolescents and young Adults | Page Nr. |
| --- | --- | --- | --- | --- |
| 1. | **Brief Name** | Maudsley Model of Anorexia nervosa Treatment for Adults (MANTRA) | Maudsley Model of Anorexia nervosa Treatment for adolescents and young adults (MANTRa) | 1 |
| 2. | **WHY: Theoretical Basis (Rationale)** | Cognitive-Interpersonal Maintenance Model of AN, considering predisposing personality traits as key vulnerability factors enhancing the risk of developing AN and maintaining factors including thinking style, social and emotional mind, pro-anorexia beliefs and unfavorable communication style of close others | Cognitive-Interpersonal Maintenance Model of AN, considering predisposing personality traits as key vulnerability factors enhancing the risk of developing AN and maintaining factors including thinking style, social and emotional mind, pro-anorexia beliefs and unfavorable communication style of close others | 4-6 |
| 3. | **WHAT: Materials used in the intervention** | MANTRA workbook for patients including 9 chapters   1. Getting started 2. Working with support 3. Nutrition 4. My anorexia: why what and how 5. Goals 6. Working towards change: 7. The emotional and social mind 8. Exploring thinking styles 9. Identity 10. Moving forward | MANTRa workbook for patients including 11 chapters   1. Introduction to MANTR-a 2. The Journey begins 3. Nobody is an island: Support from others 4. Physical health and nutrition 5. My anorexia 6. Treatment goals 7. Emotional and social relation ships 8. Thinking styles 9. Social Media 10. Identity 11. The flower of life | 10-11 |
| 4. | **Processes used in the intervention** | Psychotherapeutic intervention with multiple behavior change methods | Psychotherapeutic intervention with multiple behavior change methods and caregiver workshops | 8 |
| 5. | **WHO: Intervention provider** | Therapists from a variety of therapeutic backgrounds who are experienced in the treatment of eating disorders and trained in MANTRA | Psychotherapists and clinical psychologists trained in cognitive behavioral therapy and MANTRa | 10 |
| 6. | **HOW: Modes of Delivery** | Individual therapy sessions are provided face-to-face with the option of including close others into 2-3 sessions | Individual therapy sessions are provided face-to-face with the option of including parents into 2-3 sessions. Parental skills training groups (SUCCEAT) for carers are provided face-to-face or online. | 8, 12 |
| 7. | **WHERE: types of locations** | Patients referred to specialist eating disorder outpatient services for adults | Patients referred to a specialist eating disorder outpatient clinic for children and adolescents | 7 |
| 8. | **WHEN and HOW MUCH: frequency, duration** | 20 – 30 weekly 50 min individual therapy sessions and 4 additional monthly booster sessions | 20 – 30 weekly 50min individual therapy sessions and 4 additional monthly booster sessions |  |
| 9. | **TAILORING: personalization of the intervention** | Ongoing monitoring of physical risk is an integral part of the treatment. | All participants receive regular medical care, i. e. monitoring of their physical health and weight gain by their case manager (child and adolescent psychiatrists or child and adolescent psychiatrists in training under supervision, psychologist), and receive nutritional consultation by a dietician. Parents are regularly included in the medical treatment provided by the case manager and are additionally invited to participate in the parental skills training “SUCCEAT” which aims to reduce caregiver burden and supports parents in caring for a person with an ED | 12 |
| 10. | **MODIFICATIONS** | n.a. | n.a. | n.a. |
| 11. | **HOW WELL: strategies to improve adherence** | Therapists attended a 2 days initial training on MANTRA; Regular weekly supervision by senior clinicians. Motivational measures are included to assess if the therapeutic style is explicitly motivational. | All therapists attend a 2 –days training workshop in MANTRA and training in motivational interviewing style in a 1.5 days’ workshop. | 10 |
| 12. | **Extent to which the intervention was delivered as planned** | n.a. | n.a. | n.a. |
